# Recent Insights into Anthocyanin Pigmentation, Synthesis, Trafficking, and Regulatory Mechanisms in Rice (*Oryza sativa* L.) Caryopsis

**DOI:** 10.3390/biom11030394

**Published:** 2021-03-07

**Authors:** Enerand Mackon, Guibeline Charlie Jeazet Dongho Epse Mackon, Yafei Ma, Muhammad Haneef Kashif, Niyaz Ali, Babar Usman, Piqing Liu

**Affiliations:** 1 State Key Laboratory of Conservation and Utilization of Subtropical Agro-Bioresources, College of Agriculture, Guangxi University, Nanning 530005, China; breedermackon@st.gxu.edu.cn (E.M.); msmackon@st.gxu.edu.cn (G.C.J.D.E.M.); mayafei@st.gxu.edu.cn (Y.M.); babarusman119@gmail.com (B.U.); 2 Key Laboratory of Plant Genetics and Breeding, College of Agriculture, Guangxi University, Nanning 530005, China; tipukashif@st.gxu.edu.cn; 3 State Key Laboratory for Conservation and Utilization of Subtropical Agro-Bio-Resources, College of Life Science and Technology, Guangxi University, Nanning 530004, China; niyazali@st.gxu.edu.cn

**Keywords:** antioxidant, anthocyanin, cyanidin-3-glucoside, black rice, transcription factor, anthocyanin vacuolar intrusion, transporters, MBW complex

## Abstract

Anthocyanins are antioxidants used as natural colorants and are beneficial to human health. Anthocyanins contribute to reactive oxygen species detoxification and sustain plant growth and development under different environmental stresses. They are phenolic compounds that are broadly distributed in nature and are responsible for a wide range of attractive coloration in many plant organs. Anthocyanins are found in various parts of plants such as flowers, leaves, stems, shoots, and grains. Considering their nutritional and health attributes, anthocyanin-enriched rice or pigmented rice cultivars are a possible alternative to reduce malnutrition around the globe. Anthocyanin biosynthesis and storage in rice are complex processes in which several structural and regulatory genes are involved. In recent years, significant progress has been achieved in the molecular and genetic mechanism of anthocyanins, and their synthesis is of great interest to researchers and the scientific community. However, limited studies have reported anthocyanin synthesis, transportation, and environmental conditions that can hinder anthocyanin production in rice. Rice is a staple food around the globe, and further research on anthocyanin in rice warrants more attention. In this review, metabolic and pre-biotic activities, the underlying transportation, and storage mechanisms of anthocyanins in rice are discussed in detail. This review provides potential information for the food industry and clues for rice breeding and genetic engineering of rice.

## 1. Introduction

Anthocyanins are water-soluble plant pigments, responsible for a wide range of attractive colors in leaves, fruits, grains, and flowers [1], with most colors being red, blue, purple, and dark purple [2]. They belong to the group of phenolic compounds derived from flavonoids which play an important biological role in plants [3]. The pigments are glycosylated (aglycone), methylated or acylated forms of anthocyanidin [4,5]. Thus far, four putative functions of anthocyanins have been reported: (1) reactive oxygen species (ROS) mediators, (2) strong antioxidants, (3) chelating agents for metalloids and metals, and (4) sunscreen and promoter of leaf turgor, mainly during nutrient shortage [6]. Some scientific studies such as animal models and human clinical trials revealed that anthocyanins have potential antioxidant and antimicrobial properties, improve visual and neurological health, and protect against various non-communicable diseases [5]. Consequently, they are considered health-promoting compounds [7] and may play a fundamental role in preventing several human diseases. Other supporting studies elucidated that anthocyanins are effective in supporting glucose homeostasis [8] in the treatment of cardiovascular diseases and diabetes [9,10], hyperlipidemia and insulin resistance in fructose-fed rats [11], and cancer and tumors [2,12,13,14]. Further studies reported that anthocyanins can reduce the levels of serum creatinine, blood urea nitrogen (BUN), renal xanthine oxidase (XOD), malondialdehyde (MDA), and nitric oxide (NO) [15], Additionally, they have a positive effect in anti-carcinogenic and anti-inflammation responses [7,16]. Having these medicinal properties, anthocyanins are often used as alternatives for food colorants as well as bioactive components in nutraceutical and traditional medicine [17]. Under biotic and abiotic stress exposure, anthocyanins sustain plant growth and development as they indirectly protect leaves from damage and maintain normal photosynthetic activity [18,19,20].

**Figure 1:**
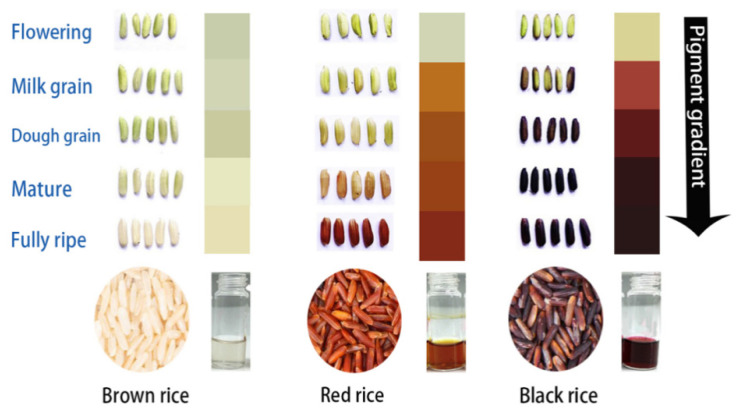
The pigment gradient in black rice compared to brown and red rice caryopses during developmental stages. Note: This figure was drawn and adapted based on the experiment conducted by Jiamyangyuen et al., 2017.

Anthocyanins have been found in different rice plant parts and most interestingly in the rice caryopsis, which leads to attractive coloration [21]. The colors of the rice caryopsis range between brown, red, purple, and black due to the varying composition and concentration of pigments [22,23,24]. The different combinations of anthocyanin and proanthocyanidin are responsible for the color differentiation (Table 1). The higher the total anthocyanin content (TAC) in the rice bran, the blacker the rice bran appears; on the contrary, the higher the amount of total proanthocyanidin (TPC), the redder the rice bran appears. The pigmentation increases as the rice caryopsis develops, and changes are observed at different developmental stages, either in the rice caryopsis or in the extracted pigment between plants accumulating anthocyanin and proanthocyanin and in non-pigmented rice (Figure 1).

The type of anthocyanin detected in black rice caryopses varies depending on the cultivar and the extraction method (Table 2). Some methods can detect both major and minor anthocyanins. High-performance liquid chromatography (HPLC) with ultraviolet-visible spectroscopy (UV–Vis) or diode array detectors (DAD) has been reported as the most applied method for separation and quantification of anthocyanins [25], and coupling this method with others can help to achieve satisfactory results. Furthermore, modern technology cannot be overlooked to achieve the most satisfactory results with more reliability.

Until now, about eighteen major types of anthocyanins have been found in rice. Among those types, four are most frequently reported, namely: cyanidin-3-glucoside (C3G), peonidin-3-glucoside (P3G), cyanidin-3-rutinoside (C3R), and cyanidin-3-galactoside [26]. Anthocyanins C3G and P3G are the most dominant. The concentration of anthocyanin in black rice caryopses also varies depending on the rice cultivar (Table 3); however, the growth environment, harvest, and storage conditions contribute to the difference as well. Moreover, it is worth mentioning that the quantity of P3G is comparatively lower than that of C3G [3,27,28,29,30,31], as the quantity of C3G ranges between 64–90% of the total anthocyanin content, and P3G accounts for 5–28% [12,32,33,34]. Nevertheless, P3G is more stable than C3G due to its methylation [1].

Increasing interest in health-promoting food has significantly generated a substantial market for potential nutritionally valuable rice [35]. Subsequently, pigmented or colored rice has received growing interest from many research programs due to anthocyanin properties, particularly its potent antioxidants and health benefits [36,37,38,39]. However, until now, the data related to anthocyanins in rice are quite limited. Different names used to identify the same candidate gene and transcriptional factor in several studies have contributed to the confusion associated with the gene network that regulates anthocyanins in rice and therefore needs to be clarified. A clear understanding of anthocyanin synthesis, trafficking, and regulation mechanism in rice will facilitate the exploration of the antioxidant properties by food industries, as well as the breeding and cultivation of black rice. Therefore, the present review has highlighted and summarized the recent insights into these areas.

**Table 1:** The concentration of anthocyanins and proanthocyanins in different types of pigmented rice.

Bran Color	Total Anthocyanin (CGE/100 g)	Total Proanthocyanidin (mg CAE/100 g)
Black	1884	78
Purple	2874	525.4
Red	8.78	716.6
Brown	3.09	4.34

CGE= cyanidin 3-*o*-glucoside equivalent, CAE= catechin acid equivalent. Note: The above table is based on the data of Goufo et al. [26].

**Table 2:** Identification of different anthocyanins in black rice cultivars.

Cultivar’s Name	Type of Anthocyanin	Methods Used	References
Baoji	Nine anthocyanins Minor Cyanidin-3,5-Glucoside, Cyanidin-3-Gentiobioside Cyanidin-3-Rutinoside Cyanidin-3-Sambubioside Major Cyanidin-3-Glucoside Peonidin-3-Glucoside Peonidin Cyanidin Cyanidin-derivative).	-High Performance Liquid Chromatography (HPLC) -Ultra-Performance Liquid Chromatography/time-of-Flight Mass Spectrometry (UPLC/Q-TOF-MS)	[15]
Longjin N°1	Four different anthocyanins Cyanidin-3-Glucoside Peonidin-3-Glucoside Cyanidin-3,5-Diglucoside Cyanidin-3-Rutinoside	-High Performance Liquid Chromatography (HPLC) -Electrospray ionization -Mass Spectrometry with diode array detection.	[1]
Okunomurasaki, Chinakuromai and Asamurasaki	Cyanidin-3-Glucoside, Peonidin-3- Glucoside Petunidin-3-Glucoside Malvidin	-High Performance Liquid Chromatography (HPLC)	[3]
Rice berry	Cyanidin-3-Glucoside Peonidin-3- Glucoside, Cyanidin, Cyanidin-3,5-Diglucoside	-Mass Spectrometric	[38]
25 rice varieties: Cabaysay, Cheng Chang, Hung Tsan, Longqing No. 3, Mitak, etc.	Cyanidin-3-Galactoside, Peonidin-3-Glucoside, Cyanidin-3-Glucoside, Cyanidin-3-Rutinoside, Cyanidin, and Peonidin	Identification based on retention times four anthocyanins and two anthocyanidins	[40]
Yunanheixiannuo	Cyanidin-3-Glucoside Cyanidin-3-Rutinoside Peonidin-3- Glucoside	-Liquid Chromatography- Mass Spectrometry (LC-MS)	[31]
Venere	Cyanidin-*O*-diglucoside, Cyanidin-3-Glucoside Cyanidin-3-Rutinoside Peonidin-3-Glucoside Peonidin-3-Rutinoside	-Liquid Chromatography- Mass Spectrometry (LC-MS)	[34]
Dongjin	Cyanidin-3-Glucoside Peonidin-3-Glucoside Cyanidin-3-Gentiobioside Cyanidin-3-Rhamnoside Cyanidin-3,5-Diglucoside Cyanidin-3-Rhamnoglucoside Peonidin-3-Rhamnoglucoside.	High Performance Liquid Chromatography (HPLC)	[41]

**Table 3:** Quantification of anthocyanin in different black rice cultivars.

Cultivar’s Name	Total Anthocyanin Content (TAC)	Major Anthocyanin	References
Venere	334 µg/g DW	Cyanidin3-*O*-glucoside (78% of TAC)	[34]
Baoji	416.92 mg CGE/g	Cyanidin 3-*O*-glucoside (76.5% of TAC)	[15]
Canada sweet rice	3276 µg/g DW	Cyanidin 3-*O*-glucoside (88% of TAC)	[42]
*O. sativa* japonica var SBR	630 µg/g DW	Cyanidin 3-*O*-glucoside (572.47 µg/g; 91.13% of TAC)	[43]
Artemide, Nerone, Venere	1404, 812 and 780 µg/g, respectively	Cyanidin3-*O*-glucoside (71%, 68%, 65% of TAC respectively)	[44]
rice berry	37 mg/100g DW	Cyanidin 3-*O*-glucoside Peonidin 3-*O*-glucoside	[38]
Yunanheixiannuo	7.5 mgCGE/g DM	Cyanidin 3-*O*-glucoside (76.8% of TAC) 85% of TAC in bran, no anthocyanin in the endosperm	[31]
Okunomurasaki, Chinakuromai, and Asamurasaki	79.5–473.7 mg/100g DM	Cyanidin 3-*O*-glucoside more than 55% of TAC	[3]
25 rice varieties: Cabaysay, Cheng Chang, Hung Tsan, Longqing No. 3, Mitak, etc.	79.5–473.7 mg/100 g DM	Cyanidin 3-*O*-glucoside ( about 82% of TAC) Anthocyanin is mainly found in bran	[40]
BIR1-3 and BJR1-3	32.4 and 160.1 mg/100g DW, respectively	Cyanidin 3-*O*-glucoside (65.62% and 89.24% respectively)	[45]

DW= Dry Weight, DM= Dry Matter, CGE= Cyanidin 3-Glucoside Equivalent.

## 2. Biosynthesis of Anthocyanin in the Rice Caryopsis and Its Relevant Mediators

**Figure 2:**
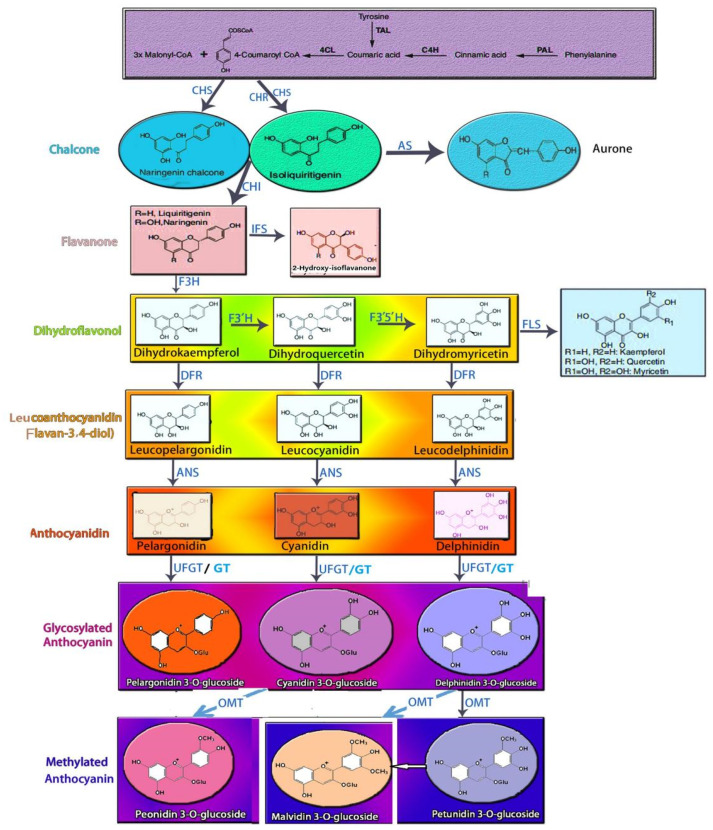
Anthocyanin biosynthesis pathways in rice.

Continuous research progress has been made in pigmented plants, highlighting the pathway of anthocyanin synthesis in rice (Figure 2). The pathway of anthocyanin biosynthesis is a branch of the general pathway of flavonoid compounds and begins with phenylalanine as a substrate [46,47,48]. This amino acid, through a series of three reactions, catalyzed successively by phenylalanine ammonia-lyase (PAL)*,* cinnamate 4-hydroxylase (C4H)*,* and 4-coumarate Coenzyme A ligase (4CL), forms another substance called 4-coumaroyl CoA. Under the control of chalcone synthase (CHS), 4-coumaroyl-CoA and 3x malonyl-CoA will react to form chalcone, which is the first committed step of the flavonoid pathway [41] and has the characteristic of flavonoid compounds [49]. With the intervention of the enzyme called chalcone isomerase (CHI), chalcone is transformed into naringenin. Oxidation of the central ring of the naringenin molecule by flavonoid 3-hydroxylase (F3H or F3′H) yields a dihydroflavonol (dihydrokaempferol). This molecule undergoes hydroxylation on the B-ring either at position 3 or 3′ and 5, with the intervention of a flavonoid 3′ or 3′-5′ hydroxylase (F3′H or F3′5′H), respectively, generating either dihydroquercetin or dihydromyricetin, which are the precursors of leucocyanidin and leucodelphinidin. Dihydroflavonols are then synthesized from flavonols catalyzed by flavonol synthase (FLS). At this point, the metabolic pathway of anthocyanins starts with one of the key enzymes called dihydroflavonol 4-reductase (DFR). Through a lack of this enzyme, the synthesis of procyanidin, anthocyanin, and pigmented rice cannot proceed. DFR is considered a key ‘late’ enzyme because it is involved in NADPH-mediated reduction of dihydroflavonols to the production of leucoanthocyanidin a flavan 3,4-diols, the immediate precursors of anthocyanidins, mainly leucocyanidin, leucodelphinidin, and leucopelargonidin [50]. 

Thereafter, the oxidation of leucoanthocyanidins catalyzed by Leucoanthocyanidin oxidase/Anthocyanin Synthase (LDOX/ANS) leads to the formation of anthocyanidins, namely, delphinidins, pelargonidins, and cyanidins [51] which are the respective precursors of purple–mauve, orange, and red–magenta anthocyanin pigments [46]. Anthocyanidin is then converted by a 3-glucosyl transferase (3GT), similar to uridine flavonoid3-o-glycosyltransferases (UFGT), to yield an anthocyanin 3-glucoside that can be further substituted by 5-glucosyl- (5GTs), rhamnosyl- (RTs), acyl- (ATs), and/or methyltransferases (MTs), resulting in ‘decorated’ anthocyanins with different colors, which are water-soluble and chemically stable pigments [52]. The decoration of anthocyanin is an important process that influences the chromatic property of the pigments. Methylation of the phenolic B ring enhances stability, reduces reactivity, increases water solubility, and subsequently reinforces its color properties [53,54]. Likewise, acylation also improves anthocyanin stability. Glycosylation affects stability through the number and position of sugar moieties on the molecule. Hence, diglucoside at C3 is more stable and stronger than monoglucoside while C5 decreases pigment intensity [55]. In pigmented rice, delphinidin is methylated to petunidin and malvidin, whereas cyanidin is methylated to peonidin [56]. Occasionally, unstable anthocyanidin can be converted by a leuco-anthocyanidin reductase (LAR) and anthocyanidin reductase (ANR) into the colorless flavan-3-ols epiafzelechin, epicatechin, and epigallocatechin [57,58]. The product will move to the vacuole with the aid of transporters, glutathione-*s*-transferase (GST), and other specific transporters localized in the vacuolar membrane [6,59].

The schematic representation of the anthocyanin biosynthesis pathway in rice was modified from Dixon et al., 2013 and Sun et al., 2018. All enzymes can be divided into two sets; however, both function as simple units. The first set is common to most flavonoid pathways, including the following genes: *PAL, C4H, 4CL, CHS, CHI,* and *F3H, F 3′H,* and *F 3′5′H*. The second set is more specific to anthocyanin biosynthesis, including genes such as *DFR, ANS,* and *UFGT*. All the structural genes encoding the enzymes involved in anthocyanin synthesis in rice have been deciphered (Table 4). In 1983, the first structural gene was identified and described in this metabolic pathway; this gene codes for chalcone synthase, pinpointed as a key element for anthocyanin synthesis [60]. The recently identified genes are on different chromosomes except chromosomes 7, 9, and 12. A mutation in these genes may lead to the production of non-pigmented rice. For instance, the *OsDFR* gene mutation had a nucleotide variation that led to a premature stop codon resulting in non-pigmented rice [61]. 

## 3. Trafficking and Accumulation of Anthocyanin in the Rice Caryopsis

**Figure 3:**
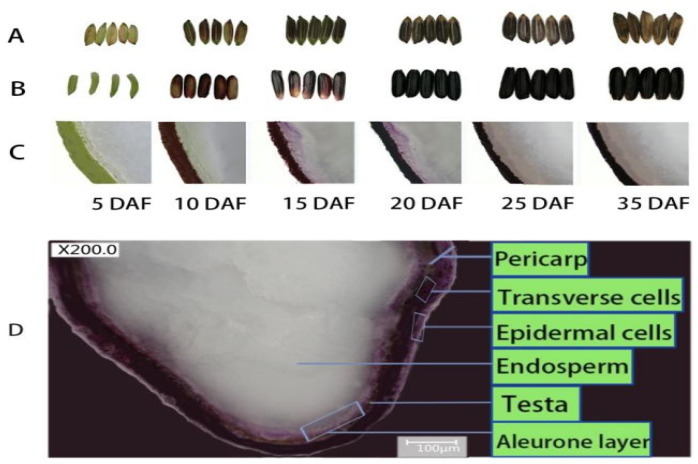
Different changes in caryopsis pigmentation during grain development in black rice. (**A**) Change in hull pigmentation of rice grain; (**B**) Evolution in caryopsis pigmentation at different developmental stage; (**C**) Longitudinal sections of caryopsis at different stages of development; (**D**) Caryopsis cross-section and super depth three-dimensional (3D) microscopic system imaging showing the pericarp, aleurone layer, and endosperm.

Anthocyanin accumulation in black rice is characterized by a gradual change in the color of the rice grain at different developmental stages (Figure 3A). The pigment gradient increases as the rice grain develop and gradually fills. The color of the caryopsis starts to be visible 8–14 days after flowering (DAF), and the caryopsis becomes black rapidly, mostly at the milk stage. The concentration of pigment is at a peak when rice is fully matured [38], 35–45 DAF (Figure 3B). This stage is the most appropriate period for harvesting because bioactive compounds are qualitatively and quantitatively well-balanced and thus more beneficial for health [31]. Likewise, the pigmentation increases with gene expression, and most of the genes are upregulated during the first 20 DAF with high activity between 7–18 DAF, while their peak is attained between 14–21 DAF [69]. 

The antioxidant capacity of colored rice originates mainly from the seed capsule [3]. Some studies revealed that more pigments are in the rice bran irrespective of whether the rice color is red, purple, or black [22,70], and these pigments are localized in the epidermal and sub-epidermal cells [71]. In black rice, anthocyanin is free in outer layers, naturally not bound to the insoluble structure but stored inside the vacuole in a large quantity [31,72]. As the rice caryopsis develops and fills-in, anthocyanin accumulates mainly inside the pericarp after 7 DAF, and then inside the testa and aleurone layer after 15–30 DAF, and almost does not accumulate in the endosperm cells [31] excluding the aleurone layer cells (Figure 3C,D).

About 97% of TAC in black rice caryopsis is found in the bran, including the pericarp, aleurone layer, and seed coat, and about 3% in the embryo, and is almost absent or only traces are found in the endosperm [26,30]. It is reported to be 30 times higher than other parts of the seed (1589.0 mg CGE per 100 g and 59.4 mg CGE per 100 g, respectively) [26]. Recent advances in molecular biology allowed the development of purple pigment in rice endosperm by a transgene stacking system [61]. The endosperm is the edible part of rice, and the recent signs of progress are of great interest. At present, many transgenic rice varieties show purple and black endosperm, having good nutritional and medical values which are of great interest to the nutraceutical and pharmaceutical industries.

**Figure 4:**
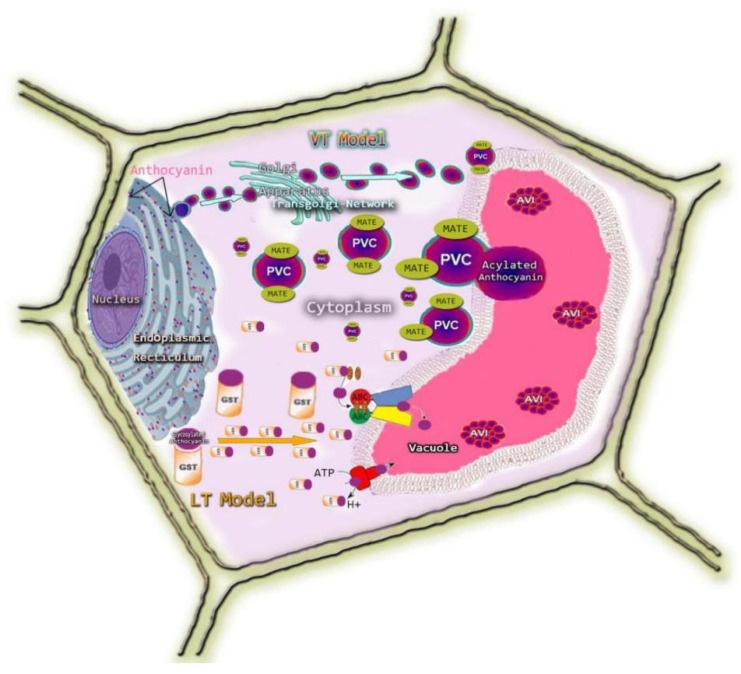
Conceptual Models of the Transport mechanisms of anthocyanin in rice.

There is no evidence that anthocyanin is transported out of the synthesizing cells. However, trafficking and accumulation are known to be intracellular. Based on the model described by Grotewold and Davies [73] and the schematic representation by Gomez et al. [74] in grapevine, here, we proposed a conceptual drawing of the transport mechanism of anthocyanin from the endoplasmic reticulum to the vacuole in pigmented rice (Figure 4). This can be helpful for further investigations. Some studies reported that anthocyanin is synthesized on the surface of the cell endoplasmic reticulum (ER) [59,75]. In the current model, we showed that in rice, once produced, the anthocyanins are transported from the site of synthesis (ER), could pass through the Golgi apparatus for acylated and methylated anthocyanin to their site of accumulation (central vacuole) in the cells of vegetative and generative organs, and be stored via vacuolar sequestration at a high concentration which gives the intensely colored chemical structure [6,59]. Previous research reported two models that attempted to explain the mechanism of anthocyanin movement from the surface of the ER to the tonoplast. The first model is vesicular transport (VT) involving the pre-vacuolar compartment (PVC), also called anthocyanic prevacuolar vesicles (APVs), which drop their cargo transported to the vacuole. In this model, the transporter is multidrug and toxic compound extrusion (MATE) [76], also called antho-mate (Anthocyanin Mate). This transporter accompanies PVC filled with anthocyanin to the tonoplast, and then the vesicle secretory pathway occurs thereafter; the anthocyanin enters the vacuole. In the VT model, anthocyanin vacuolar intrusion (AVI) in the vacuole occurs as an autophagy mechanism of intact vesicles [77]. The second model is ligandin transportation (LT), which involves ligandins that escort anthocyanin products to the vacuole and sequester these into anthocyanic vacuolar intrusion (AVIs), mostly when the concentration of anthocyanin in the cytoplasm is too high. In this model, anthocyanin first binds to a suitable transporter (ligandin) and diffuses through active transport until it reaches the tonoplast. The suitable transporter is glutathione-s-transferase (GST), which is located in the cytoplasm and is associated with the ER [78,79]. It acts as a binding protein that escorts anthocyanins from the ER to the tonoplast. As the anthocyanin reaches the tonoplast, it penetrates the vacuole through the ATP-Binding Cassette (ABC). 

We schematically present two models to explain the transport mechanisms of anthocyanin in rice. In the LT model, GST carries mainly glycosylated anthocyanin. These anthocyanins penetrate the vacuole through ABC. In the VT model, the MATE transporter carries mainly acylated anthocyanins, which are transported in the PVC. The anthocyanin is stored as AVI and diffuses inside the vacuole. 

In rice, two anthocyanin transporters have been characterized (Table 5). As a member of the ATP-dependent, proton-gradient-independent transporter ABC superfamily [80], multidrug resistance-associated protein (MRP) is involved in the vacuolar sequestration of potentially toxic metabolites and other secondary metabolites [59]. A multidrug resistance-associated protein (MRP) was identified to be involved in anthocyanin transport in rice [81]. The investigation revealed that the MRP-encoding gene called *OsMRP15* is an orthologous gene of maize anthocyanin transporter *ZmMRP3* [82]. *OsMRP15* co-expresses with OsC1 and OsB1 transcription factors (TFs) in different tissues and organs except the embryo to promote a membrane-bound transporter protein OsMRP15 required for vacuole uptake of anthocyanin in rice [81]. The Clustered Regularly Interspaced Short palindromic Repeats, CRISPR-associated proteins9 (CRISPR/Cas9) guided mutations in *OsMRP15* resulted in green leaves, whereas its wild type had purple leaves [83]. Likewise, *OsGSTU,* a homolog of the maize Bronze2 [84] mutation, induced green leaves in a rice line with purple leaves, confirming the role of these genes in anthocyanin accumulation in rice leaves [83]. Thus, *OsMRP15* and *OsGSTU* may play a central role in anthocyanin accumulation by facilitating the transport and sequestration of anthocyanin in the vacuole of pigmented rice.

Furthermore, it is believed that besides GST and ABC transporters, MATE plays a role in rice anthocyanin transport and accumulation. The MATE transporters are distinct from any other known multidrug resistance protein transporters because of the 12 predicted transmembrane I helices and serve as transporters in the accumulation and sequestration of the flavonoid and secondary metabolites in the vacuole [76,85]. 

The six rice MATE proteins (OsMATE7, OsMATE34, OsMATE33, OsMATE3, OsMATE39, OsMATE16) and five characterized transporters of various glycosides (VvAM3, VvAM1, SIMATE, AtFFT, MtMATE2) involved in the transport of anthocyanin into vacuole were classified in the same sub-cluster [76]. Thereafter, these six rice MATE proteins were presumptively involved in anthocyanin mediation in rice. MATE proteins play a noticeable role in rice for detoxification and are implicated in increasing the tolerance of pigmented and some non-pigmented rice against abiotic stress through metabolite, alkaloid sequestration, hormone, and organic acid transport [85]. Although the intracellular characterization of MATE proteins in black rice and their involvement in anthocyanin transportation is not yet elucidated, it is suggested that GST-mediated, ABC transporter-mediated, and MATE transporter-mediated transport may co-exist in the same cells and may engage in the anthocyanin transport mechanism of pigmented rice. Deductively, we believe that in this process, the LT model would prevail over the VT model because the main anthocyanin produced in rice is the glycosylated form (C3G).

## 4. Regulation of Anthocyanin in the Rice Caryopsis

### 4.1. The MYB-bHLH-WD40 (MBW) Complex and Activation of the Anthocyanin Biosynthesis Pathway in Rice

In most cases, the ternary complex MYB-bHLH-WD40 is a player in activating the anthocyanin biosynthesis structural genes in plants. In this complex, the MYB TFs belong to subgroup 6 of the R2R3-MYB family and carry a highly conserved signature motif. The MYB proteins of the MBW complex bind to DNA and are believed to be the main components of the MBW complex that activates the target genes. The activity of MYB in controlling anthocyanin biosynthesis is specific. Usually, rice has multiple copies (paralogues) of this gene, which confer anthocyanin production in different patterns or cell types. It is the activity of the MYB TFs that normally determines the amount of anthocyanin production by specific cells, and so differences in rice color intensity and pattern are usually attributable to differences in the expression of the MYB TFs in the MBW complex. The basic helix-loop-helix (bHLH) partners in the MBW complex may have many independent functions and are not present exclusively in the cells in which anthocyanin is synthesized. As such, their functions are not only limited to the regulation of anthocyanin biosynthesis but also to the regulation of proanthocyanidin biosynthesis, as well as the control of vacuolar Ph and seed coat morphology. In plants, bHLH proteins belong to multigenic families [86], encompassing members in rice (*O. sativa*). The broad range of biological functions for the bHLH partners in the MBW complex implies that they are not the determinants of anthocyanin in different cell types. Contrary to the arguments mentioned above, the *Kala4* gene, which is the rearrangement of the promoter region of *OsB2* (a bHLH protein gene), is expressed ectopically in the pericarp of the black rice and activates the transcription of anthocyanin biosynthetic genes [87]. This demonstrated the specified role of the bHLH partners in the MBW complex. The WDR proteins of the MBW complex are the scaffolding proteins and probably interact directly with the bHLH proteins, besides binding to the DNA. The structural genes in anthocyanin biosynthesis are regulated differently in dicots and monocots [88]. The dicot structural genes in anthocyanin biosynthesis can be divided into groups, composed of early biosynthesis genes (EBG) and late biosynthesis genes (LBG). The EBGs include the major genes encoding chalcone synthase (CHS), chalcone isomerase (CHI), and flavanone 3-hydroxylase (F3H), while the LBGs include the important genes encoding dihydroflavonol 4-reductase (*DFR)* and anthocyanin synthase (ANS) [89]. In dicot plants, the expressions of EBGs and LBGs are controlled separately [90]. In a monocot plant such as pigmented rice, all the structural genes (*OsPAL*, *OsCHS, OsCHI, OsF3H, OsDFR, OsANS/LDOX*, and *OsUFGT/Os3GT*) and Os*ADR* function as a simple unit activated by a complex [91]. Some components of the MBW complex have been characterized in rice (Table 6), elucidating the regulatory mechanism of anthocyanin synthesis in rice. 

The possible regulation of the MBW complex has been deciphered. Two novel regulators *OsBBX14* and *OsHY5* TFs were reported to be localized in the nucleus with transcriptional activation activity that triggered anthocyanin biosynthesis regulatory genes *OsC1* and *OsB2* [96]. The C-terminal of *OsBBX14* is a functional region for transcriptional activation. The physical interaction of the basic leucine zipper (Bzip) domain of *OsHY5* with the second B-box of *OsBBX14* induced *OsC1* expression. High expression of *OsBBX14* is further worth noting, associated with the high transcript level of *OsHY5* in the pigmentation of black rice seeds [96]. This indicates that *OsBBX14* and *OsHY5* could act in a coordinated or separate way to directly activate regulatory genes *OsC1* and *OsB2*. The expression of *OsBBX14* increased significantly and gradually with seed maturation from 15 DAF to 30 DAF [96]. In addition to anthocyanin accumulation, *OsBBX14* is surprisingly involved in photo-morphogenesis, hypocotyl length, and chlorophyll accumulation [97]. This could be a new foundation for regulatory mechanisms in pigmented rice. Apart from regulating the ternary MBW complex, some mediators (proteins) can solely regulate the expression of the structural genes involved in the anthocyanin biosynthesis of rice. The mediator OsP1 activates the upstream biosynthetic genes (*OsCHS, OsCHI, OsF3H)* for anthocyanin biosynthesis [91].

### 4.2. Black Rice Caryopsis Pigmentation: From Classical Genetics to Molecular Genetics 

The rice caryopsis consists of the pericarp, testa, embryo, and endosperm. Since the anthocyanin is mainly deposited in the pericarp of the caryopsis, genetic study of the black rice caryopsis pigmentation mainly focuses on the pericarp. The pigmentation of the pericarp has been widely studied, and the results showed three phenotypes of the F_2_ populations derived from the crosses between black rice and white rice. The segregation ratio was 9:3:4 for the three phenotypes, black pericarp, brown pericarp, and white pericarp [98,99]. The presence of genes and their status (whether recessive or dominant) greatly affect the intensity of pericarp coloration in rice genic analysis [99]. The purple pericarp is governed by the two genes purple pericarp b (*Pb* synonym *Prp-b*) and purple pericarp p (*Pp*, synonym *Prp-a*). The product of *Pb* has been revealed to be responsible for pigment deposition in the pericarp of brown grain, whereas *Pp* amplifies the accumulation of pigmentation leading to purple grain. The copy number of the *Pp* allele determines the intensity of the purple coloration, and the *Pp* allele is incompletely dominant to the recessive *pp* allele [99]. Thus, the simultaneous presence of these two dominant genes, *Pb* and *Pp,* results in the purple coloration of rice pericarp, while the presence of *Pb* and the absence of *Pp* result in brown coloration. In addition, *Pb* is epistatic to *Pp* and *pp* of rice pericarp; the absence of *Pp* results in white coloration. These two dominant genes, *Pb* and *Pp,* are mapped to chromosomes 4 and 1, respectively [98,99]. The *Pb* locus harbors two genes named *Ra* and *bhlh16.* The former is a homolog of the Myc TF *Lc* gene regulating anthocyanin biosynthesis in maize, and the latter is a homolog of the *TT8* gene, which is an Myc TF gene controlling proanthocyanidin synthesis in the pericarp of *Arabidopsis thaliana* [100]. *Ra* and *OsB1* have been reported to have some common functions in anthocyanin synthesis [92,100,101], and a 2-bp (GT) deletion found in exon 7 of the *Ra* gene in all purple pericarp compared to white pericarp was suggested to be the origin of purple pericarp [100]. In another recent study, it was revealed that the black pericarp pigmentation is governed by the interaction of key activator loci for anthocyanin (KALA), named *Kala1, Kala3,* and *Kala4* [95]. Quantitative trait loci (QTL) mapping identified *Kala1* between the SSR markers RM 7405 and RM 7419 on chromosome 1, *Kala3* between the SSR markers RM15191 and RM 3400 on chromosome 3, and *Kala4* between SSR markers RM 1354 and RM 7210 on chromosome 4 [95]. The *Kala 4* region harbored three alleles, namely, *Os04g0557200, Os04g0557500,* and *Os04g0557800,* coding for bHLH proteins [87]. Subsequent analysis showed that among these alleles, *Os04g0557500* plays a key role in anthocyanin pigmentation in rice pericarp. Thereby, this allele was believed to be *Kala4*. *Os04g0557500* has the same function as *OsB2,* whereas *Os04g0557800* corresponds to *OsB1* previously identified in Chr 4 at a purple leaf locus *(Pl^W^)* [92]. Since *Kala 4* and *Pb* are mapped to the same region, *Pb* is suggested to be an allele of the *Kala4* locus [87]. This study further demonstrates that *Kala4,* a bHLH gene, undergoes rearrangement in the promoter region, expresses ectopically in the pericarp, and activates anthocyanin synthesis genes, such as chalcone synthase and dihydroflavonol-4-reductase, leucoanthocyanidin reductase, and leucoanthocyanidin dioxygenase, producing the pigments in the pericarp [87]. Therefore, *Kala4* and *OsB2* may be synonymous since they have the same function, while *pb* is likely to be the same gene as *Ra* and *OsB1* [101]. However, both *OsB1* and *OsB2* identified in the Pl locus co-segregated to enhance anthocyanin in leaves [92], while *Kala4* (*Os04g0557500)* with a rearrangement in the promoter region could induce the black pericarp coloration [87]. The chromosomal region of *Kala1* includes the *Rd* locus coding for the DFR enzyme, which is regulated by a member of the Kala4 locus for anthocyanin synthesis. Thus, *Rd* may be synonymous with *Pp,* which is believed to be *Kala1.* It is likely that *Kala3* is synonymous with *MYB3* [95].

With the advent of modern molecular techniques, e.g., QTL mapping, next-generation sequencing, and microarray, researchers have more and more tools to elucidate the pigmentation of colored rice. QTL identification technology was used to locate the QTLs underlying anthocyanins in rice, detecting that the seven QTLs for anthocyanin are located on chromosomes 1, 3, 7, and 10 [27].

### 4.3. Molecular Regulation of Anthocyanin Production in the Rice Caryopsis

To better understand the anthocyanin gene expression in black rice, the 135K *O. sativa* microarray was used to evaluate the expression of genes after the heading stage. Eighty-two TFs genes classified into 12 groups were found to be associated with anthocyanin. Besides, the comparisons between the white and black cultivars revealed that twelve hypothetical unknown genes were involved in anthocyanin biosynthesis [41]. Furthermore, a combination of whole-genome resequencing, RNA-sequencing (RNA-seq), microarray, and reverse-transcriptase polymerase chain reaction (RT-PCR) was used to identify anthocyanin biosynthesis-related genes; finally, nine anthocyanin-related genes were verified to be related to the regulation of anthocyanin biosynthesis and/or metabolism [96,102].

Two kinds of genes are involved in rice anthocyanin biosynthesis, the structural genes encoding the enzymes in the biosynthesis pathway, and the regulatory genes encoding TFs. The regulatory genes control the expression levels of structural genes by the action of the TF, influencing the intensity and pattern of anthocyanin biosynthesis. In black rice, two classes of regulatory genes, namely, the R/B gene family encoding Myc bHLH proteins and the C1/Pl gene family encoding Myb-type R2R3 proteins, have been reported to control the accumulation and deposition pattern of anthocyanin [100,103]. Some rice regulatory genes have been isolated and characterized (Table 6).

**Figure 5:**
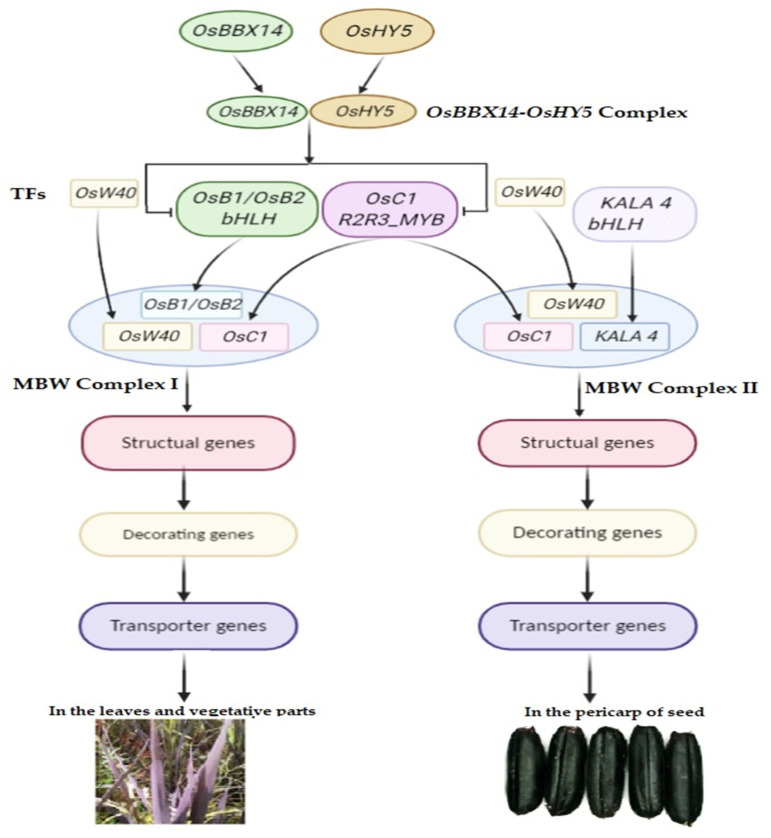
Regulatory mechanism and transcriptional factor interactions in black rice.

The structural genes (*OsCHS, OsCHI, OsF3H, OsDFR, OsANS/LDOX,* and *OsUFGT/Os3GT*) function as a single unit activated by a complex MBW [41,69] that regulates anthocyanin in black caryopses (Figure 5). In this complex, the bHLH TF protein is encoded by the *Kala4*/*OsB2 gene* located on chromosome 4 [87,95] and the R2R3-MYB TF encoded by candidate C gene or OsC1 located on chromosome 6 (Os06g0205100) [94,104,105,106]. The WD40 protein acts as a scaffolding molecule, assisting the proper activity of other proteins, and its relevant gene *OsWD40* is located on chromosome 2 and has been cloned and characterized [61]. However, *OsC1* has been revealed as a potential activator of *DFR* and *ANS* involved in the anthocyanin biosynthesis in other plant organs, mainly hull pigmentation [107], and *OsB2* in a tissue-specific manner [87,92]. Therefore, there is still a possibility that further genes and variants thereof might be highlighted.

A mutation in regulatory genes may lead to changes in pigmented rice. The *OsB1* gene mutation had a 2-base insertion, causing amino acid substitutions in the functional domains and a premature stop codon, thus producing a non-functional protein. Sakulsingharoj and colleagues elucidated that a 2 bp (GT) insertion in exon 7 of *OSB1*, which along with a 1 bp deletion of a guanine nucleotide in exon 8, results in a threonine for methionine substitution at position 64, resulting in a white grain phenotype [104]. Likewise, the *osb2* allele of *OsB2* had a single-base insertion causing a premature stop codon, which resulted in the loss of function of the gene [61]. The MYB family shares a central role in the coordination and activation of sets of specific genes involved in anthocyanin pathways [4]. MBW encoding-genes have been identified and are well-known. Some environmental factors can affect their regulation process in anthocyanin synthesis, including temperature, light, and nutrient influences on the MBW complex [108,109,110].

This overview summarizes the TFs involved in anthocyanin pigmentation and their interaction to regulate anthocyanin synthesis in black rice. In this process OsBBX14, a B-box BBX TF, interacts with OsHY5 to form a complex that triggers OsC1, OsB1, and OsB2 TFs. These TFs engage with other MYB and bHLH TFs to form different types of MBW (complex I & II), which activate the expression of structural genes for the formation of anthocyanin. OsB1-OsW40-OsC1 will form complex I, which regulates anthocyanin synthesis mainly in leaves and other vegetative parts, whereas, Kala4-OsW40-OsC1 binds to complex II that regulates anthocyanin mainly in the pericarp of seed (Figure 5). The anthocyanins are decorated, transported by the transporter proteins, and stored into the vacuole of cells [61].

Recent high-throughput sequencing technologies include transcriptome analysis, genome re-sequencing, fine mapping, and cloning, unraveling the main candidate networks directly involved in the coordination of anthocyanin formation in rice. Coordinating sets of genes and TFs are responsible for phenotypic diversity in pigmented rice. Several authors endeavored to associate variant genes with the variation of phenotype in pigmented rice. Thereafter, this has been extensively studied using different advanced molecular biology techniques, and the basic genes and QTLs for anthocyanin expression of several rice parts have been found (Table 7).

## 5. Enhanced Anthocyanin Accumulation in Rice Caryopses through Genetic and Environmental Modulation

### 5.1. Genetic Engineering Approach

Anthocyanin is a source of pharmaceuticals, food additives, flavors, and industrial biochemical components [5,17]. An increase in anthocyanins in rice is a great challenge, and the amount and types of anthocyanins in rice vary according to cultivars and growth conditions [27].

The transgenic approach is a powerful tool to investigate regulatory genes and genetic engineering in plants [118]. The anthocyanin pigmentation process in rice has been useful to understand the role of several genes involved in the anthocyanin biosynthesis pathway and the enhancement of anthocyanin in the endosperm. A white rice japonica cultivar, Hwa-Young, was transformed with maize *C1/R-S* regulatory genes using the promoter of a rice prolamin gene. The result showed the expression of the gene in the endosperm and revealed numerous kinds of flavonoid compounds including anthocyanin endosperm [119]. Anthocyanin pigmentation provides an excellent system that can be used to study the regulation of genes in higher plants. Reddy et al. [22] used a transgenic approach to reveal the function of anthocyanidin synthase (ANS), one of the four dioxygenases (DOX) of the flavonoid biosynthetic pathway in the synthesis of anthocyanidins from leucoanthocyanidins. The result suggested that ANS may act directly on different flavonoid substrates of DOX reactions. Furthermore, Kiwahigashi et al. [120] induced the expression of *OsB2* after treatment with methyl jasmonate (MeJA) and 2,6 dichloro-isonicotinic (DCINA), resulting in the accumulation of anthocyanin in rice. Subsequently, chemical treatment of transgenic plants increased the activity of *OsB2* and *OsANS,* leading to a high accumulation of anthocyanin in rice [120]. Similarly, Sakulsingharoj et al. [121] cloned the Myc-type bHLH gene OsB2 from the black rice variety Khum and transformed it into white rice Nipponbare and Taichung 65 using *Agrobacterium tumefaciens*. The result showed that transgenic rice up-regulated the expression of structural genes, both EBG (F3H) and LBG (DFR 4, ANS), concomitantly [121]. This result supports other evidence that in rice EBG and LBG are not regulated separately. More recently, Zhu et al. [61] developed a high-efficiency vector system containing two regulatory genes and six structural anthocyanin-related genes driven by endosperm-specific promoters to engineer purple endosperm rice (Zijingmi). This result provided a new rice collection for germplasm and validated the successful use of a transgene stacking system for the biosynthesis pathway. These results are a significant achievement for anthocyanin production in rice.

### 5.2. Environmental Modulation

Environmental conditions are crucial in rice production and can affect the productivity of black rice [122,123,124,125,126]. Anthocyanin is a highly unstable pigment; the stability and intensity of pigments are influenced by multiple prevailing conditions [55,127,128]. In rice, several factors including temperature, light intensity, pH, drought, minerals, heavy metal ions and salt, enzymes, sugar structure, and sugar metabolites have been reported to affect anthocyanin biosynthesis or degradation directly and indirectly.

#### 5.2.1. Chemical Treatment

Anthocyanin structure is essential to maintain its stability. Highly hydroxylated anthocyanin is less stable than acylated and methylated anthocyanin. The larger the hydroxyl group, the bluer the color becomes [128,129]. The stability of anthocyanin can be enhanced by direct chemical modification of its structure. The stability of anthocyanin of black rice variety can be enhanced through acylation with succinate anhydride (OSA). The acylated anthocyanin was less affected by pH, light, high temperature, with high color retention and density; however, it lost its solubility in water [130].

#### 5.2.2. Temperature

Anthocyanin biosynthesis is mostly affected by temperature. Suitable temperature increases anthocyanin synthesis while extremely high or low temperature reduces the accumulation of anthocyanin and induces degradation of pigments [131,132,133,134,135]. Inhibition of the expression of anthocyanin activator genes or enhancement of the expression of the repressor genes was observed when plants were exposed to high temperature, leading to decreasing anthocyanin accumulation [136,137]. In the rice caryopsis peculiarly, high temperature ≥ 35 °C especially during grain filling has an inhibitory effect on anthocyanin accumulation, whereas low temperature between 22–27 °C enhances anthocyanin biosynthesis. High temperature induces an alteration in the expression of CHS, F3H, DFR, and ANS genes in rice and hinders anthocyanin synthesis, resulting in decreasing accumulation of anthocyanin in the rice caryopsis. A daily mean temperature of about 32 °C compared to that of 22 °C can result in a reduction of more than 20% in the amount of anthocyanin during grain filling [138]. Kim and co-workers [69] reported that the expression level of *CHS, F3H, DFR, ANS,* and *AN5* of the Heugjinju black rice cultivar was 200–500-fold lower for plants grown under 21 °C compared to those grown under 27 °C. Thus, the growing altitude may impact the anthocyanin content and color density of pigmented rice; an appropriate altitude for a cultivar can help to enhance the accumulation of anthocyanin. A black rice variety G60 increased its anthocyanin content about 2-fold when grown at an altitude of 1360 m as compared to when grown at an altitude of 76 m. Consequently, the color of the caryopsis was darker at high altitude compared to low altitude. This might be attributed to the temperature effect that was different between the two altitudes [139]. Thermal stress enhances peroxidase enzyme activity and a high level of H_2_O_2_, resulting in the degradation of anthocyanin [140]. Thus, under high temperature (≥35 °C), the application of peroxidase inhibitors could help to maintain metabolic activities and sustain anthocyanin accumulation in pigmented rice.

#### 5.2.3. Light

It is well known that light is a great stimulus for plant growth, and light intensity positively correlates with the level of phenolic compounds such as anthocyanin [132,141]. Although in rice no such experiment has been conducted, there is some evidence about the effect of light on anthocyanin. For instance, Huang and co-workers [141] elucidated the effect of ample sunlight on the fruit peel of blood orange and purple pummelo. The experiment conducted in the orchard demonstrated that the amount of anthocyanin in the orange fruit peel was reduced by 20-fold in bagged oranges compared to the oranges under natural light. Subsequent analysis showed that the active promoter of *Ruby1*, a key activator of anthocyanin biosynthesis, was light-inducible [141]. Likewise, Chan and colleagues [142] highlighted the effect of irradiance and light intensity on anthocyanin production in *Melastoma malabathricus* and found that light exposure positively regulated the accumulation of anthocyanin whereas shade or a dark environment repressed it. Higher accumulation of anthocyanin was observed with moderate light intensity (301–600 lx). The cultures exposed to 10 days of continuous darkness showed the lowest pigment content, while the cultures exposed to 10 days of continuous irradiance showed the highest pigment content [142]. Moreover, the synergistic effect of low temperature and light has been demonstrated in grapes, and many anthocyanin-related genes were upregulated independently under both conditions [143]. This condition may also influence anthocyanin biosynthesis in pigmented rice.

#### 5.2.4. Salinity or Salt Stress

Salt stress is among the most severe environmental stresses that cause the accumulation of ROS damage in plants [144,145]. Anthocyanin accumulation is a protective mechanism for major anthocyanin productive plants under stress [146]. In rice, the amount of anthocyanin in leaves increased during the first four days of NaCl treatment. The anthocyanin concentration was negatively affected by about 25% when the rice plant was exposed to a concentration of 150 Mm of NaCl for six to eight days [147]. The expression patterns of some genes, *OsPAL, OsCHS, OsDFR,* and *OsANS,* were more influenced during salt stress and correlated with the anthocyanin variation in the leaf. The higher the total anthocyanin in leaves (deep purple leaf color) the less the plant is affected (growth parameter is less affected). This indicates that higher anthocyanin in the leaf can significantly reduce salt stress in a plant [147].

#### 5.2.5. Nutrient Supply

Nutrients are essential for plant growth in all stages of plant development. Some minerals such as calcium, magnesium, iron, manganese, and copper can form a complex with anthocyanin in plant cells [148]. It has been shown that improving Mg uptake in rice can form a metalloid co-pigment complex for stabilizing anthocyanin, thus increasing the concentration in different parts such as leaves and the pericarp. Mg promotes the expression level of *OsPAL, OsANS, OsDFR, OsF3H,* and *OsCHS* [149]. This result agrees with Sinilal and co-workers [148], who reported that Mg^2+^ acts as a co-enzyme regulator in anthocyanin biosynthesis that could sustain a high level of anthocyanin in the rice pericarp. Later on, Yamuangmorn and colleagues [150] reported that nitrogen fertilizers applied to rice increased anthocyanins in the leaf and shoot but had no effect on the anthocyanin concentration in the grain [150]. These environmental conditions, if used carefully, may be a good approach to easily increase anthocyanin in pigmented rice. However, more investigation is still needed.

## 6. Conclusions

To ensure food and nutritional security, rice has been considered the best staple food. The world’s population is expected to reach 9.7 billion by 2050; food and energy demands should be a great challenge [151]. New strategies should be adopted to increase food production and nutritional values [105]. As a rich antioxidant, anthocyanin manipulation in rice could be a good approach to enhance rice quality and nutritional value.

Over the last decade, anthocyanin has been considered an attractive and rich functional nutrient in food and has attracted many researchers due to its antioxidant properties. Significant progress has been made to understand its potential health benefit; some key genes involved in the regulatory network and transcriptional factors have been reported, and biosynthesis pathways have been revealed. However, spatial–temporal anthocyanin accumulation and various phenotypes make the understanding of anthocyanin synthesis in pigmented rice more complex. In this regard, this review highlights numerous studies leading to different results useful for a better understanding of the synthesis mechanism and transportation of anthocyanin in pigmented rice for their better handling.

About eighteen types of anthocyanins are found in the rice caryopsis, among them, cyanidin-3-glucoside (C3G) and peonidin-3-glucoside (P3G) are the most abundant. Anthocyanins are spatially–temporally expressed differently in rice parts, and biosynthesis involves the regulatory ternary complex R2R3-MYB, bHLH and W40 transcriptional factors, which deferentially activate structural genes for enzyme synthesis. They are synthesized on the surface of the endoplasmic reticulum of cells and are transported to the vacuole of a large range of cells and tissues of vegetative and generative organs at a high level through vesicular transport and ligandin transportation.

However, this field needs to be deeply studied to identify the gaps in relevant mechanisms, enabling the enhancement of anthocyanin in rice. For instance, future investigations should address the functional characterization of the genes involved in the storage mechanism of anthocyanin in rice and gene regulation mechanisms encoding for anthocyanin transporters. Powerful tools such as gene-editing tools, e.g., CRISPR/Cas9, can be utilized to modulate transcriptional factors to enhance anthocyanin in rice endosperm. Besides, the conserved regulatory mechanism in the expression of a gene such as histone modification, non-coding RNA, and DNA methylation, could be used to highlight the biological processes of anthocyanin in rice. The environmental factors (temperature, drought, disease tolerance, heavy metal stress, hormone, nutrients, etc.) that can influence the accumulation of anthocyanin could be addressed to promote anthocyanin production in rice. Whole-genome resequencing could accelerate and allow the discovery of novel allelic variants, with unknown genes subsequently used to develop markers. The development of good quality markers can be helpful to support marker-assisted selection for rapid breeding. Indeed, there are flourishing opportunities for breeders to develop productive pigmented varieties for their antioxidant properties.

## Figures and Tables

**Table 4:** Anthocyanin biosynthesis-related genes in rice.

Groups	Protein	Gene Name	Locus ID	Reference
**Phenylalanine Pathway Structural Genes**	Phenylalanine Ammonia-Lyase (PAL)	*OsPAL*	*Os02g0627100*	[62]
	Cinnamate 4-Hydroxylase (C4H)	*OsC4H*	*Os05g0320700*	[61,63]
	4-Coumaroyl CoA Ligase (4CL)	*Os4CL*	*Os02g0177600*	[64]
**Anthocyanin Pathway Structural Genes**	Chalcone Synthase (CHS)	*OsCHS*	*Os11g0530600*	[65]
	Chalcone Isomerase (CHI)	*OsCHI*	*Os03g0819600*	[65]
	Flavanone 3-Hydroxylase (F3H)	*OsF3H*	*Os04g0662600*	[65]
	Flavanone 3′-Hydroxylase (F3′H)	*OsF3′H*	*Os10g0320100*	[65]
	flavonoid 3′-5′ hydroxylase (F 3′5′H)	*OsF3′5′H*	*Os03g0367200*	[61]
	dihydroflavonol 4-reductase (DFR)	*OsDFR*	*Os01g0633500*	[65]
	Anthocyanidin synthase (ANS)	*OsANS*	*Os01g0372500*	[65]
	Leucoanthocyanidin reductase (LAR)	*OsLAR*	*Os03g0259400b*	[64]
	anthocyanidin reductase (ANR)	*OsANR*	*Os04g0630800*	[66]
**Decorating Genes**	Anthocyanin 3-*O*-glycosyltransferases	*Os3GT*	*Os06g0192100*	[67]
	Anthocyanin 3′-*O*-Methyltransferase	*Os3′MT*	*Os08g0157500*	[68]

**Table 5:** Anthocyanin transporter genes in rice.

Protein	Gene Name	Locus ID	Description	Reference
Glutathione-*S*-transferase U 34	*OsGSTU34*	*Os10g0395400*	Thioredoxin fold domain-containing protein (characterized)	[83]
Multidrug resistance-associated proteins 15	*OsMRP15*	*Os06g0158900*	Similar to Multidrug-resistance associated protein 3 (characterized)	[83]
Multidrug and toxic compound extrusion 7	*OsMATE7*	*Os02g0821600*	Similar to mate efflux family protein (uncharacterized)	[76]
Multidrug and toxic compound extrusion 34	*OsMATE34*	*Os08g0562800*	Similar to Transparent testa 12 protein (uncharacterized)	[76]
Multidrug and toxic compound extrusion 33	*OsMATE33*	*Os08g0550200*	Multi antimicrobial extrusion protein MatE family protein (uncharacterized)	[76]
Multidrug and toxic compound extrusion 3	*OsMATE3*	*Os01g0766000*	Multi antimicrobial extrusion protein MatE family protein (uncharacterized)	[76]
Multidrug and toxic compound extrusion 39	*OsMATE39*	*Os10g0195000*	Multi antimicrobial extrusion protein MatE family protein (uncharacterized)	[76]
Multidrug and toxic compound extrusion 16	*OsMATE16*	*Os03g0626700*	Multi antimicrobial extrusion protein MatE family protein (uncharacterized)	[76]

**Table 6:** Anthocyanin production regulatory genes in rice.

Protein	Gene	Locus ID	Reference
bHLH TF	*OSB1*	*Os04g0557800*	[92]
	*OSB2*	*Os04g0557500*	[92]
	*Kala4*	*Os04g0557500*	[87]
	*OSRc*	*Os07g0211500*	[93]
R2R3-MYB TF	*OsC1*	*Os06g0205100*	[94]
*OsMYB3*	*Kala 3*	*Os03t0410000*	[95]
*WD40* repeat	*OsWD40*	*Os02g0682500*	[61]

**Table 7:** QTL associated with various phenotypes responsible for anthocyanin in rice.

Phenotype	Name	Location	Fine Mapping	Reference
Colored apiculus	C-gene	RM19552-RM19565 or RM 111-RM253	C Locus was found to be about 59.3 kb between the SSR markers RM111 and RM 253 with a genetic distance of 0.7 and 0.4, respectively.	[111,112]
Purple apiculus	*Pa-6*	RM19556—RM19561 with 0.2–0.3 cM respectively	Pa-6 is located 41.7 kb between L02 and RM 19561 containing 11 ORFs of which ORF8 is associated with this trait.	[111]
Red apiculus	*OSC*	An interval of 70.8 kb bounded Dcap13-RM 19561	OSC is located to an interval of 70.8 kb bounded by Dcap13-RM 19561 and contains 10 ORFs of which ORF8 consists of two introns and three exons. It contains a 10-bp deletion in the third exon, causing a frame-shift mutation and loss of function of the encoded protein.	[113]
Black hull	*Bh1*	RM6629 and SNP marker SNP6-1	*Bh1* was fine- mapped on the long arm of Chr4 in an interval of 24.2 kb between RM 6629 and SNP6-1.	[114]
Purple pericarp	*Pb*	An interval of 25 kb of RID3 and RID4	Pb was first mapped downstream of SSR RM3820 on Chr4 and between RID3 and RID4 after saturation with indel and CAP markers	[100]
Purple leaf	*Plr4*	Recessive gene, Two putative candidates *Os04g0577800* and *Os04g0616400*	The purple leaf gene was located on Chr4 to about 27.9–31.1 Mb.	[115]
Purple stigma	*Ps-4(t)*	RM253, RM111 and RM6917	The *Ps-4(t)* gene was mapped on Chr 6 at 2.5 cM, 0 cM and 4.4 cM, respectively, from RM_253_, RM_111_ and RM_6917_	[116]
Purple leaf sheath	Purple leaf sheath	SSR markers RPM8 and RPM11	Purple leaf sheath was first mapped to the short arm of Chr6 between RPM5 and RM402 with a genetic distance 1.1 and 10.3 cM, respectively, and then narrowed to an interval of 153 kb between RPM8-RPM11	[117]

## Data Availability

No datasets were generated or analyzed during the current study.

## Abbreviations

4 CL	4-Coumaroyl CoA Ligase
ABC	ATP-Binding Cassette
AM	Anthomate
ANR	Anthocyanidin reductase
ANS	Anthocyanin synthase
APV	Anthocyanic pre-vacuolar
AVI	Anthocyanic vacuolar intrusion
Bhlh	Basic helix-loop-helix
BUN	Blood Urea Nitrogen
C3G	Cyanidin-3-glucoside
C3R	Cyanidin 3-Rutinoside
C4H	Cinnamate 4-hydroxylase
CAE	Catechin acid equivalent
CGE	Cyanidin 3-*o*-glucoside equivalent,
Chr	Chromosome
CRISPR/Cas9	Clustered Regularly Interspaced Short palindromic Repeats/CRISPR-associated protein9
DAD	Diode array detectors
DAF	Days after flowering
DM	Dry matter
DW	Dry weight
EBG	Early biosynthesis genes
ER	Endoplasmic reticulum
F 3′5′H	Flavonoid 3′-5′ hydroxylase
F3H	Flavanone 3-hydroxylase
FLS	Flavonol synthase
GST	Glutathione-*S*-Transferase
KALA	Key Activator Loci for Anthocyanin
LAR	Leuco-Anthocyanidin Reductase
LC-MS	Liquid Chromatography-Mass Spectrometry
LDOX	Leucoanthocyanidin dioxygenase
LT	Ligandin transportations
MATE	Multidrug and toxic compound extrusion
MDA	Malondialdehyde
NO	Nitric Oxide
ORF	Open reading frame
OsBBX14	Oryza sativa B-box14
OsHY5	*Oryza sativa* Hypocotyl 5
P3G	Peonidin-3-glucoside
PAL	Phenylalanine ammonia-lyase,
PVC	Pre-vacuolar compartment
QTL	Quantitative trait loci
ROS	Reactive oxygen species
TAC	Total anthocyanin content
u-HPLC	Ultra-High-performance liquid chromatography
UFGT	Uridine flavonoid 3-*o*-glycosyltransferases
UVD	Ultraviolet detection
UV-vis	ultraviolet-visible spectroscopy
VLB	Vesicle-like bodies
VT	Vesicular transport
